# Outbound medical tourists: The interplay of perceived quality, length of stay, group-size, post-visit destination image and revisit intention

**DOI:** 10.1371/journal.pone.0267755

**Published:** 2022-05-10

**Authors:** Imran Rahman, David S. Martin, Sijun Liu

**Affiliations:** Department of Nutrition, Dietetics & Hospitality Management, College of Human Sciences, Auburn University, Auburn, AL, United States of America; St John’s University, UNITED KINGDOM

## Abstract

Using halo effect as the underlying theory, we examined how perceived quality of medical care influenced components of post-visit destination image (infrastructure, attraction, value for money, and enjoyment) and how each component influenced Bangladeshi outbound medical tourists’ revisit intentions. Additionally, we examined how these relationships varied based on their length of stay (LOS) and travel-group size (TGS). Results showed a significant positive effect of the perceived quality of medical care on all four components of the post-visit destination image. Except for enjoyment, all three components had a significant positive influence on revisit intentions. All the proposed relationships were supported for medical tourists with higher LOS and TGS. However, for medical tourists with low LOS, the perceived quality of medical care did not influence value for money. Furthermore, value for money and enjoyment did not significantly influence revisit intentions for medical tourists with low LOS and TGS.

## Introduction

Medical tourism refers to patients leaving their resident country and crossing a border to receive medical care [1, 2]. The medical tourism market is estimated to produce $65 to $87.5 billion in revenue, which is generated from the 20 to 24 million medical tourists across the world who spend more than $3000 on average per visit [3]. While much of the current research has focused on medical tourism emanating from a developed country, patients from many developing countries, such as Bangladesh, continue to travel abroad for better medical care and represent an essential part of the overall medical tourism market [4]. For instance, the Government of India reported that in 2017, as many as 221,751 Bangladeshi medical tourists traveled to India for various medical treatments [5]. In 2019, before COVID-19 made international travel difficult, Bangladeshis accounted for 57.5% of international medical tourists to India [6]. Medical tourism is also one of the fastest-growing tourism sectors globally [7]. It has become an essential part of several countries’ tourism economies [8]. Increases in the size of the medical tourism marketplace and rising levels of competition for the medical tourists’ dollars has led practitioners to seek new ways to attract potential medical tourists and motivate those same tourists to revisit the same destination in the future, whether that be for future medical treatments or as a more traditional tourism destination [9]. As the medical tourism marketplace continues to develop and mature, both practitioners and researchers require a better understanding of specific factors that impact the revisit intentions of these specialized tourists to maintain a competitive advantage in this growing market.

While previous research has investigated multiple areas within medical tourism, the majority of these studies have focused on first-time destination choice or visit intention [10–12], with only a limited number of research studies evaluating the revisit intentions of medical tourists [7, 13]. Moreover, much of the previous research has used evaluations of the overall destination image instead of looking into specific components of the tourists’ post-visit destination image [14]. While understanding the impact that overall destination image has on the revisit intentions of medical tourists is certainly important, a more nuanced approach to the individual factors that make up destination image and how each of these may or may not impact the revisit intentions of medical tourists is warranted. Practitioners and marketers need this level of detail to better understand what factors drive re-visit intentions and provide important information into where money and effort should be directed by medical tourism destinations, especially in light of the growing competition for medical tourism dollars globally.

Using halo effect as the underlying theoretical foundation, this study investigates how the perceived quality of medical care affects the post-visit destination image components. Variables considered include infrastructure, attractions, value for money, and enjoyment. Additionally, we measured how each of these components, in turn, influences medical tourists’ revisit intentions and how these relations vary depending on the length of stay and travel group size. The following research questions are addressed in this study:

1. How does the perceived quality of medical care influence components of the post-visit destination image of medical tourists?
2. How do the components of post-visit destination image influence the revisit intentions of medical tourists?
3. How do the relationships between perceived quality of medical care, components of post-visit destination image, and revisit intention vary based on medical tourists’ length of stay and travel-group size?

## Literature review

### Understanding destination image

Destination Image (DI) is defined as perceptions that individuals have about a destination [15]. More simply put, DI is how a tourist both thinks and feels about a tourism destination. As noted by many, DI is currently, and has been, a primary focus of researchers within the tourism literature [16, 17]. According to [18], DI has been worthy of scholarly focus since the 1970s but started to gain traction as an essential research topic as early as the 1990s [19]. Much of this focus has been derived from the need to distinguish one tourist destination from another, especially in an increasingly competitive global marketplace [19]. Classifying and organizing DI has primarily been based on the cognitive-affective theoretical model of image formation. Cognitive evaluation refers to consumers’ specific beliefs and knowledge about a destination, while affective imagery relates to a consumer’s feelings about the destination [20, 21]. In combination, both cognitive and affective images form the overall image of a destination [22].

[23] postulated that three types of DI exist: organic, induced, and complex images. An organic image is formed through exposure to stimuli from non-tourism-related media. Examples of this include television shows or movies, which feature images from a specific location. An induced image is based on specialized marketing or advertising material, usually prepared by or in concert with a specific tourist destination. Noted for their intentionality, these materials are often produced by a destination marketing organization (DMO). They are focused on promoting particular tourism-related activities, aka trying to convince potential tourists to visit a specific location or country. It should also be noted that both organic and induced images are formed before an actual visit by a tourist to an actual destination [24]. Complex images differ from both induced and organic images in that they are based on the individual’s direct experience on-site [23, 25]. These complex images are significant, especially when considering the importance of revisit intentions to destinations.

[25] developed an eighteen-item scale to measure tourists’ post-visit complex DI. Their scale includes four components–infrastructure, attraction, value for money, and enjoyment. Infrastructure refers to the physical elements, both public and private, needed to access the destination and enjoy it safely, such as police service, airports, road networks, hospitals, etc. [26]. The attraction of a tourist destination is the perceived ability of a destination to meet the needs of tourists through its desirable features and attributes [27]. Cultural and natural attractions, recreation facilities, and events are considered major facets of visitor attraction of a destination [28]. The value for money of a destination refers to the price-quality relationship when purchasing a tourism product such as food, accommodation, tour, souvenir, etc. [29]. Lastly, enjoyment refers to the process of deriving a psychological benefit such as pleasure from visiting a destination or from participating in a touristic activity in that destination [30]. Infrastructure, attraction, and value for money represent the cognitive images, while enjoyment represents the affective component [25]. These four factors, sometimes in different forms, are found to represent destination image in the extant tourism literature (e.g. [31, 32]).

Destination image has been a reliable and accurate antecedent of tourism satisfaction, destination choice, and post-visit behavioral intentions [33, 34]. This may be due to the influence DI plays in forming expectations about a location, which is then used as a point of comparison both during and after a visit has occurred [35]. Repeat visitors are a much sought-after market segment for many, including countries that have invested heavily in becoming a medical tourism destination [36]. Repeat visitors incur much lower marketing costs than first-time visitors [37], stay longer at a destination [38], spread positive word of mouth [37], and participate in destination-specific consumptive activities more meticulously [39]. More recently, [14] conducted a meta-analysis of 35 articles and found a strong connection between positive complex DI and behavioral loyalty.

[23] found that expectations of non-visitors exceeded the actual performance reported by visitors, indicating a disconnect between what the destination projects in its promotional and marketing efforts and the actual delivery of those same products and services. They also found that the overall DI held by non-visitors significantly differed from that of actual visitors. Such differences have primarily been attributed to tourist destinations’ marketing and promotional efforts, which may or may not reflect the real experiences that occur once on-site. Medical tourism destinations have indeed engaged in similar types of promotions, with even specific hospitals devoting significant resources to developing websites promoting their services to potential medical tourists [40]. Because of the inherent differences between organic/induced and complex images, it may be beneficial to examine the factors which contribute to complex images, as we do in this study, to gain a complete understanding of revisit intentions. A recent study by [41] on Chinese medical tourists visiting Malaysia found that the overall image of Malaysia as a medical tourism destination positively influences revisit intentions. In this study, we look into the components of complex destination image of the country to determine which components influence revisit intentions.

### Perceived quality of medical care and the halo effect

[42] noted that medical services themselves are challenging for a consumer to evaluate, especially before their application. This is because medical services are high in credence properties, which are attributes that make them difficult to assess without a unique level of skills and background knowledge specific to medical treatment [43]. Furthermore, previous research has indicated that medical tourists consider the perceived quality of medical care as the most critical attribute for destination selection [10, 44], and according to [45], higher levels of perceived quality of medical care from a medical facility leads to a better corporate image of that same medical provider.

[13] and [46], using a purposive sample of Bangladeshi medical tourists to India, showed that healthcare service quality positively influences medical tourists’ satisfaction, which in turn affects their loyalty, i.e., revisit intention and intention to recommend. However, the satisfaction and loyalty measured in the aforementioned studies were specific to the medical facility, not for the destination as a whole. Because medical services themselves can only be evaluated after they have been performed, it is vital to examine how the perception of the quality of medical care impacts the medical tourist’s DI and how these images also impact revisit intentions. We anticipate that higher levels of perceived quality of medical care provided by the medical facility can lead to improved DI due to a positive halo effect.

Initially conceptualized by psychologist Edward L. Thorndike in 1920, the halo effect is a popular theory used in marketing and consumer behavior studies. A more common understanding of the halo effect is that positive impressions in one area can spill over positive opinions or feelings in other areas [47]. For example, customers are often biased toward certain products because of favorable or unfavorable experiences with other products made by the same company or brand. In the tourism literature, [48] have shown that tourists’ DI of a country positively influences perceptions of products originating from that country. Beyond tourism, in a study of perceptions of wine attributes, [49] confirmed the positive halo effect of natural corks when compared with screw caps and synthetic corks. In their study, wine thought to have been poured from a bottle with a natural cork closure registered significantly higher ratings on appearance, bouquet, taste, and overall quality compared with wines closed with screw caps or synthetic cork closures, creating a positive halo effect for wines enclosed with the superiorly perceived natural cork. Similarly, [50] showed via a sensory experiment involving 203 consumers that wines primed as genetically modified received less desirable evaluations on appearance, aroma, and taste relative to conventional samples, confirming a negative halo effect of gene technology in wine production.

As a widespread and widely researched cognitive bias, the halo effect has not been applied in the context of medical tourism. By applying the halo effect to medical tourism, the perceived quality of medical care that a patient receives from a medical provider might also influence his/her overall evaluation of the destination. Similarly, a negative experience with a medical provider may also taint the medical tourist’s views about the destination itself and other related ancillary components, such as the infrastructure, the attractions, value for money, and even enjoyment. Based on these ideas, we propose the following hypotheses:

*Hypothesis 1*: Perceived quality of medical care positively influences the components of post-visit DI: A)Infrastructure; B)Attraction; C)Value for money; D)Enjoyment.

*Hypothesis 2*: The components of post-visit DI: A)Infrastructure; B)Attraction; C)Value for money; D)Enjoyment positively influence revisit intention.

### The impact of length of stay and travel-group

It is anticipated that the proposed hypotheses in this study may vary based on medical tourists’ length of stay (LOS) and travel-group size (TGS). LOS as a variable has received considerable attention in tourism research, with most studies focusing on its determinants [51–54] and a few on its outcomes [55, 56]. [57] suggested a positive but diminishing relationship between LOS and tourism expenditures. [58] found that repeat visits influenced LOS negatively. [23] found that long-term tourists had better images of social opportunities, attractions and infrastructure, food, and friendly people than short-term tourists in the Rio Grande Valley in Texas, USA. According to [56], LOS significantly increased the desirability of a destination and knowledge related to it. According to [59], long-term tourists (travelers who spent seven nights or more) were significantly more satisfied with the quality of tourism service providers, the perceived efficiency during their vacation, and the cost of the trip than short-term tourists. Therefore, it is highly likely that tourists, who stay longer at a destination, may develop a more positive image of the destination, contributing to higher revisit intentions. According to [55], higher LOS significantly increased the positive experience of nursing care received at a hospital. Therefore, the positive halo effect that spills from the quality of medical care to the components of DI might be stronger for long-term medical tourists.

Travel-group size (TGS), or in other words, the number of people traveling with a patient, can have an impact on the overall medical tourism experience. According to Lovelock and [60], those who travel alone were less likely to engage in a range of normally expected holiday behavior in a medical tourism context. According to [61], a larger TGS indicates more time spent on the tourism component of medical tourism because leisurely travel is more predominant when friends and family are present. A survey of medical tourists in South Korea found that when medical tourists are accompanied by family or caregivers, tourism and tourist facilities become highly essential [62]. In general, traveling companions in a medical tourism context help in recovery, moral support, and the motivation to behave in a more conventionally touristic way [60]. Therefore, the perceived quality of medical care, DI, and revisit intention might be higher when TGS is larger.

Based on the related literature review, it is safe to assert that LOS and TGS are expected to impact our proposed model. More specifically, we anticipate that the strength of most of our proposed relationships will be stronger for long-term medical tourists and for medical tourists with a larger travel group.

## Methodology

This study was approved by the Auburn University IRB Board prior to data collection. A self-report survey was developed using Qualtrics. Data were collected using telephone surveys. The convenience sample consisted of 331 Bangladeshi medical tourists who traveled abroad for medical tourism between January 2015 and March 2018. We contracted with a local consumer panel company to solicit responses from outbound Bangladeshi medical tourists. The consumer panel company managed a database of Bangladeshi outbound tourists who traveled abroad for medical tourism. A total of 978 Bangladeshi medical tourists were contacted via telephone. The response rate, therefore, was 33.8%. The company was paid $500 (about $1.50 per completed survey). Data was collected in March 2018. It took the consumer panel company three weeks to collect the 331 completed responses. The questions in the survey were not tailored to any particular destination or medical facility. Respondents were asked to only consider their last international outbound medical tourism trip when answering this survey.

Destination image was measured using [25] 18-item scale. The scale has four components–infrastructure, attraction, value for money, and enjoyment. The four components of destination image–infrastructure, attraction, value for money, and enjoyment, validated in [25], were treated as four variables in the proposed model of this study. Perceived medical quality was gathered from the previously validated three-item scale used by [63] and [7]. Revisit intention was measured using three items adapted from [64]. The questions were measured on a 5-point Likert scale, ranging from strongly disagree (1) to strongly agree (5). Demographic information was collected via questions about gender, age, education, marital status, income, country visited, travel-group size, length of stay, and type of procedure. The survey was translated to Bengali, the official language of Bangladesh, by a professional native-speaking translator from a professional translation service organization. The survey was back-translated to English by another native-speaking professional translator as a form of validation for accurate wording. Participants answered the phone survey in 5 to 15 minutes. On average, it took participants 8 minutes to answer the 33-question survey, as reported by the consumer panel company.

Data were analyzed in SPSS and AMOS. Descriptive analysis, reliability analysis, confirmatory factor analysis, path analysis, and multi-group analyses were conducted to examine the data. Data were further broken down into multiple groups to test any effects of length of stay (LOS) and travel-group size (TGS) on the proposed hypotheses. LOS was broken down into two groups: medical tourists who stayed six nights and less and medical tourists who stayed seven nights and more. We used this breakdown based on prior studies [59, 65, 66]. TGS was divided into two groups: medical tourists who traveled with three people and less and those who traveled with four people or more. This division was based on our data as most medical tourists traveled with 3 or 4 people in our sample.

## Results

We received 331 completed responses from the consumer panel company. Nine responses were discarded for being incomplete. The participants for the current project were overwhelmingly male (91%), below the age of 51 (94.1%), married (74%), and well-educated (72.7% had at least a bachelor’s degree). 52.5% of respondents went for relatively simple procedures such as a routine check-up, and 60.6% of respondents stayed longer than seven nights. Interestingly, 100% of the participants traveled with at least one companion, with 48.8% of the participants traveling in a group of 4 or more. More than half of the sample traveled to India, which shares a border with Bangladesh and is accessible via air and road networks. Additionally, it should be noted that language and cultural barriers in India are very low for Bangladeshi natives due to the historical connections between these two countries and the popularity and availability of products and services in Bangladesh that originate from India. Other Asian countries such as Thailand (20.5%) and Singapore (20.2%) were also popular choices for Bangladeshi medical tourists. Table 1 presents the demographic profiles of the respondents.

**Table 1:** Respondents’ demographic profile.

	N	%
**Gender (N = 322)**		
Male	293	91
Female	29	9
**Age (N = 322)**		
18–30	97	30.1
31–40	149	46.3
41–50	57	17.7
51+	19	5.9
**Marital Status (N = 322)**		
Single	54	16.8
Married	239	74.2
Separated	16	5
Divorced	9	2.8
Widowed	4	1.2
**Education (N = 322)**		
Did not complete High School	1	0.3
High school or equivalent	14	4.3
Some college	73	22.7
Bachelor’s degree	114	35.4
Master’s degree	92	28.6
Advanced, Professional or Doctorate degree	28	8.7
**Income (N = 320)**		
$0 to $24,999	294	91.3
$25,000 to $49,999	28	8.7
**Country Traveled (N = 322)**		
India	165	51.2
Thailand	66	20.5
Singapore	65	20.2
England	8	2.5
Malaysia	7	2.2
Japan	5	1.6
China	3	0.9
Dubai	2	0.6
Saudi Arabia	1	0.3
**Travel-group Size (N = 322)**		
Solo	0	0
2	2	0.6
3	163	50.6
4	113	35.1
5	33	10.3
6 and above	11	3.4
**Length of Stay (N = 322)**		
2–3 nights	39	12.1
4–6 nights	88	27.3
7–9 nights	84	26.1
9+ nights	111	34.5
**Procedure Type (N = 322)**		
General Check-up/Diagnosis	169	52.5
Dental	7	2.2
Cosmetic	34	10.6
Surgery	89	27.6
Cancer/Tumor Treatment	21	6.5
Gender Modification	2	0.6

Descriptive analysis and reliability analysis were computed in SPSS. All variables demonstrated strong Chronbach’s Alpha values (0.82 and above), confirming the reliability of our measures. Please see Table 2 for the details.

**Table 2:** Descriptive statistics and reliability analysis.

Measure	Mean	SD	Cronbach’s Alpha
Perceived Quality of Medical Care	4.54	.70	.89
Destination Image	3.98	.57	.91
Revisit Intention	3.89	.84	.89
Image of Infrastructure	4.28	.85	.93
Image of Attraction	4.01	.65	.82
Image of Value for Money	3.68	.79	.84
Image of Enjoyment	3.96	.74	.89

The four-factor destination image scale of [25] was validated in this study through confirmatory factor analysis (CFA) using maximum likelihood estimation. All factor loadings, except for one, were above .60, the recommended minimum suggested by most researchers [67, 68]. Accordingly, one of the items under enjoyment, “city is a novel travel destination”, was deleted for having a factor loading of 0.57. All the remaining factor loadings ranged from 0.60 to 0.89. [69] suggested the most important measures to report are the model chi-square, CFI, RMSEA and SRMR. The resulting CFA yielded the following fit statistics. *χ*^2^ = 208.39; df = 81; p < .001; CFI = .94; RMSEA = .09; SRMR = .06. Therefore, the four-factor model as suggested by [25] was deemed acceptable.

Path analysis was undertaken using AMOS. The results of the analysis in Table 3 showed that all hypotheses, except H2D_,_ were supported. Therefore, the image of enjoyment was not found to influence the revisit-intention of medical tourists significantly. Perceived quality of medical care has significant positive effects on all four components of the post-visit DI. Image of infrastructure, image of attraction, and image of value for money had significant positive effects on revisit intention.

**Table 3:** Results of path analysis.

Hypotheses	Paths	*Β*	C.R.	Results
H1A	Perceived Quality of Medical Care→Image of Infrastructure	0.72***	18.71	Supported
H1B	Perceived Quality of Medical Care→Image of Attraction	0.65***	15.39	Supported
H1C	Perceived Quality of Medical Care→Image of Value for Money	0.25***	4.55	Supported
H1D	Perceived Quality of Medical Care→Image of Enjoyment	0.45***	8.90	Supported
H2A	Image of Infrastructure→Revisit Intention	0.49***	10.86	Supported
H2B	Image of Attraction→Revisit Intention	0.27***	6.02	Supported
H2C	Image of Value for Money→Revisit Intention	0.16***	4.17	Supported
H2D	Image of Enjoyment→Revisit Intention	0.03	0.71	Not Supported

***P<0.001

**P<0.01

*P<0.05.

The respondents were divided into sub-samples to examine the interactions based on length of stay (LOS) and travel-group size (TGS). This part of the study was exploratory, and the researchers anticipated that there will be some variations in our results depending on LOS and TGS.

LOS was divided into two groups. Group 1 consisted of participants who stayed six nights or less, resulting in a sample of 127 participants. Group 2 comprised participants who stayed seven nights or more, resulting in a sample of 195 participants. A few studies have determined that the average LOS of medical tourists is around 6–7 nights [65, 66]. Descriptive statistics and reliability analysis of the two groups are presented in Table 4.

**Table 4:** Descriptive statistics and reliability analysis—Length of stay.

	Length of Stay<6 Night; N = 127			Length of Stay>6 Nights; N = 195		
Measure	Mean	SD	Cronbach’s Alpha	Mean	SD	Cronbach’s Alpha
Perceived Quality of Medical Care	4.49	.75	.86	4.57	.68	.91
Destination Image	3.76	.50	.85	4.12	.56	.94
Revisit Intention	3.68	.75	.89	4.03	.87	.89
Image of Infrastructure	4.04	.88	.90	4.43	.80	.94
Image of Attraction	3.81	.61	.71	4.15	.64	.86
Image of Value for Money	3.29	.77	.75	3.93	.70	.85
Image of Enjoyment	3.90	.96	.94	3.99	.56	.80

We carried out path analysis on the two groups. For group 1, all hypotheses were supported except H1C, H2C, and H2D. For group 2, all hypotheses were supported. The results are presented in Table 5.

**Table 5:** Path analysis results—length of stay.

#	Hypotheses	(Length of Stay≤6 Nights); N = 127		(Length of Stay>6 Nights); N = 195	
		*Β*	Result	*β*	Result
H1A	Perceived Quality of Medical Care→Image of Infrastructure	0.61***	Supported	0.82***	Supported
H1B	Perceived Quality of Medical Care→Image of Attraction	0.49***	Supported	0.78***	Supported
H1C	Perceived Quality of Medical Care→Image of Value for Money	-0.07	Not Supported	0.49*	Supported
H1D	Perceived Quality of Medical Care→Image of Enjoyment	0.27**	Supported	0.68***	Supported
H2A	Image of Infrastructure→Revisit Intention	0.48***	Supported	0.52***	Supported
H2B	Image of Attraction→Revisit Intention	0.28***	Supported	0.16**	Supported
H2C	Image of Value for Money→Revisit Intention	0.04	Not Supported	0.16***	Supported
H2D	Image of Enjoyment→Revisit Intention	-0.10	Not Supported	0.14**	Supported

***P<0.001

**P<0.01

*P<0.05.

Travel-group was divided into two groups. Group 1 consisted of three people or less, resulting in a sample of 165 participants, and group 2 consisted of more than three people, resulting in a sample of 157 participants. Groups were divided to ensure each group had an adequate number of samples to analyze. Descriptive statistics and reliability analysis are represented in Table 6 after the division of the travel group.

**Table 6:** Descriptive statistics and reliability analysis—Travel group.

	Travel-group≤3 People; N = 165			Travel-group>3 People; N = 157		
Measure	Mean	SD	Cronbach’s Alpha	Mean	SD	Cronbach’s Alpha
Perceived Quality of Medical Care	4.71	.61	.90	4.36	.76	.91
Destination Image	4.10	.56	.92	3.86	.55	.90
Revisit Intention	4.09	.73	.86	3.69	.90	.90
Image of Infrastructure	4.41	.86	.94	4.14	.83	.91
Image of Attraction	4.15	.63	.81	3.87	.65	.81
Image of Value for Money	3.78	.79	.85	3.58	.79	.82
Image of Enjoyment	4.05	.70	.90	3.86	.78	.87

For the travel group representing three people and less, all hypotheses were supported except for H2C and H2D. For the larger travel group representing more than three people, all hypotheses were supported. Table 7 presents the findings.

**Table 7:** Path analysis results—Travel-group.

#	Hypotheses	(Travel-group≤3 People); N = 165		(Travel-group>3 People); N = 157	
		*β*	Result	*Β*	Result
H1A	Perceived Quality of Medical Care→Image of Infrastructure	0.65***	Supported	0.78***	Supported
H1B	Perceived Quality of Medical Care→Image of Attraction	0.57***	Supported	0.69***	Supported
H1C	Perceived Quality of Medical Care→Image of Value for Money	0.27***	Supported	0.18*	Supported
H1D	Perceived Quality of Medical Care→Image of Enjoyment	0.35***	Supported	0.49***	Supported
H2A	Image of Infrastructure→Revisit Intention	0.71***	Supported	0.46***	Supported
H2B	Image of Attraction→Revisit Intention	0.16**	Supported	0.19**	Supported
H2C	Image of Value for Money→Revisit Intention	0.06	Not Supported	0.22***	Supported
H2D	Image of Enjoyment→Revisit Intention	-0.29***	Not Supported	0.21***	Supported

***P<0.001

**P<0.01

*P<0.05.

## Discussion

**Fig 1:**
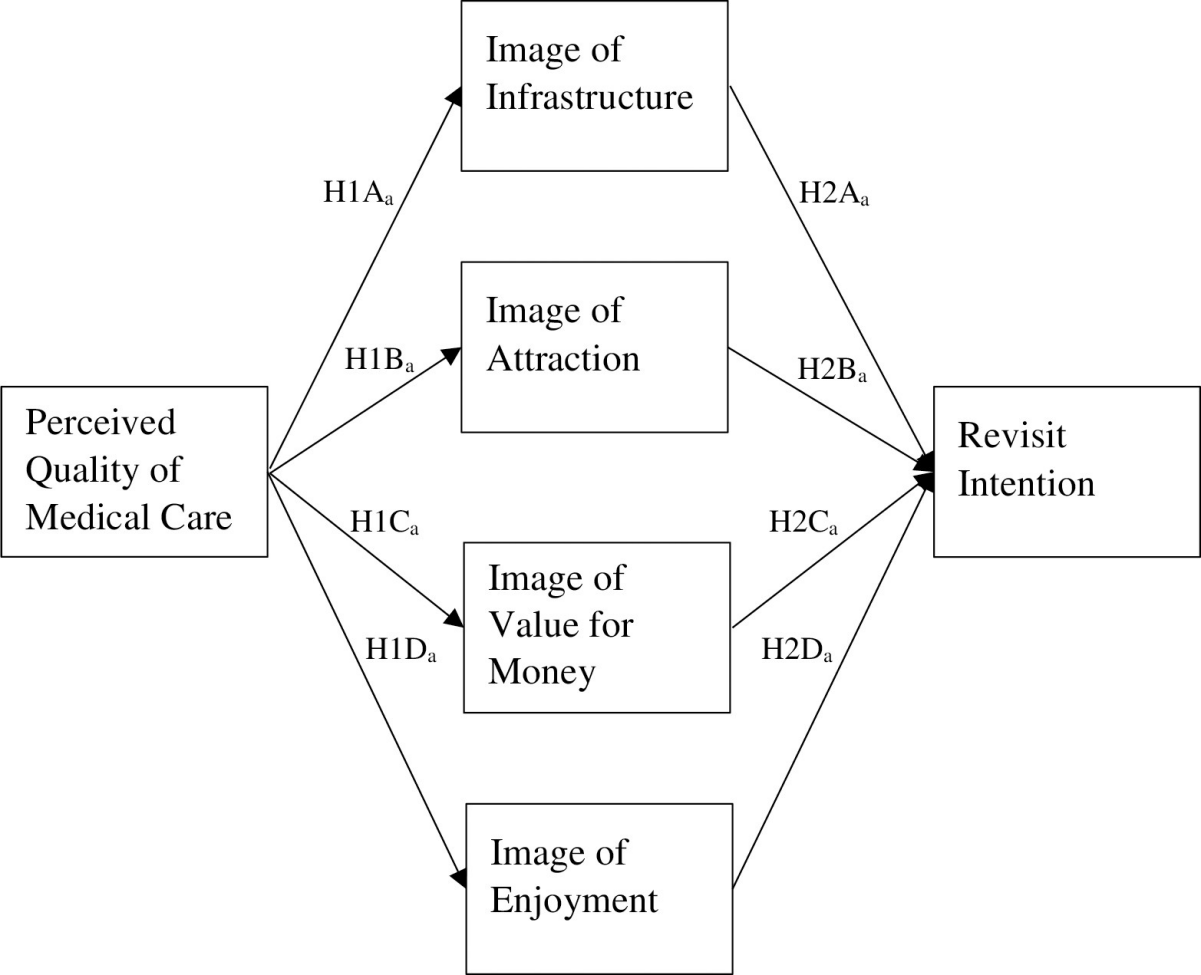
Conceptual model.

Regarding the path analysis of the overall model (See Fig 1), the perceived quality of medical care had a significant positive effect on the images of infrastructure, attraction, value for money, and enjoyment. These findings indicate support for a halo effect between the perceived quality of medical care and all four components that make up destination image. Therefore, our results suggest that the perception of the quality of medical care received does spill over into the overall evaluation of destination image made by medical tourists. As noted by previous researchers [70–72], a medical tourist’s primary motivation for medical tourism is often driven by the desire to secure high-quality medical care. This seems especially likely for medical tourists departing from Bangladesh, where high costs, unethical practices in the medical supply chain, poor service, improper treatment, long waiting times, and a lack of skilled personnel have been identified as issues within the Bangladeshi health care system [46, 71, 73, 74]. Based on the previous work of [75], which found that tourists often use environmental cues to form and guide their perceived image of a destination, the researchers propose that perceived quality of medical care is taking on the role of an environmental cue for Bangladeshi medical tourists, which then shaped their overall formation of DI and significantly impacted all four individual components of destination image.

Three out of the four components of DI (image of infrastructure, attraction, and value for money) positively influenced revisit intention, which leaves the image of enjoyment as the only variable to not influence revisit intention. This finding may be best explained by the primary motivation of the travel itself (medical treatment vs. for pleasure) and the general aversion that many consumers have when engaging with medical treatment [76]. This reticence to engage with medical treatments, coupled with the high credence levels associated with medical care, may lead to a perception by patients that future visits are either unnecessary or not worth the investment, even if the quality of medical care was perceived favorably. This finding is noteworthy in that it highlights the importance of the three other variables that make up DI (infrastructure, attraction, and value for money) in the revisit intentions of medical tourists.

Interestingly, when examining the proposed model after the data set had been divided based on length of stay (seven nights or more vs. less than six nights), differences between the two groups emerged, as well as differences between each group and the overall model. For respondents that stayed more than six nights, every path proposed (See Fig 1) was supported, including the path from enjoyment to revisit intention. While further work is needed in this area, this finding is similar to the previous work of [55], who found that increased length of stay significantly increased the positive experience of nursing care received at a hospital. Increased lengths of stay may also improve the ability of the medical personnel and the patient to form a more personal connection and build trust, which may also increase overall healing by the patient. As noted by [77], "The development of trust in the physician leads to a proper patient-doctor relationship and is part of the healing process." This increased time and trust would also potentially allow the patient to realize the importance and necessity of the treatment(s) being offered and follow-up visits. Finally, increased length of stay may provide more opportunity for the patient to engage with more pleasurable aspects of the tourism experience, instead of shorter visits that may be only focused on the medical-based experiences.

The results differed more drastically for patients who stayed for six nights or less, with the path between perceived medical care and value for money not being supported. The researchers feel that this finding requires more research to better understand why this sub-group path was not supported. One possible explanation may be the direct and indirect costs associated with travel to a foreign country for medical treatment vs. the time spent in the country. In other words, the time-value proposition for shorter trips, which in theory provides less opportunity for engagement in the more hedonistic features of the tourism destination and less time to developing a trusting relationship with the medical provider (and potentially less overall healing), may be perceived by the patients to be of inferior value. This may also help to explain why the paths between value for money and enjoyment to revisit intentions were also not supported for patients who stayed for six nights or less. More research into these specific areas is warranted, as it seems that patients with shorter visits are evaluating their experiences differently than patients who stayed for more than six nights.

Regarding travel group size, subsets were created using groups of three or less and groups of four or more. Similar to the findings for individuals who stayed more than six nights, groups of four or more had supported paths between all of the variables proposed in Fig 1. For groups of three travelers or less, the paths between value for money, enjoyment, and revisit intentions were not supported, which mirrors the findings for a length of stay that is six nights or less. This finding seems to support previous results by [61], where a larger travel group size indicates more time spent on the tourism component of medical tourism. Similarly, [62] found that when family or caregivers accompany medical tourists, tourism and tourism facilities become of great importance. Again, the researchers feel that more research is needed to help identify the underlying issues here, but fewer caregivers accompanying the patient may mean that each caregiver has more individual work to do to care for their traveling patient. This, in turn, may impact the patients’ ability to enjoy more traditional tourism activities and impact their perception of the value they receive in relation to the direct and indirect costs associated with their travel. In a similar vein, overworked caregivers who have not been able to engage in the more pleasurable aspects of the medical tourism experience may also influence the patients’ perceptions.

### Practical implications

The current study indicates that medical tourists are not a monolithic group. Instead, they have essential subgroups that evaluate their experiences as medical tourists differently. Specifically, shorter stays and smaller groups of travelers are different in determining their revisit intentions compared to longer stays (seven or more nights) and more extensive travel groups (more than three people). For groups of three people or less, or for travelers who are staying for six nights or less, both value for money and enjoyment did not support revisit intentions. When medical tourists and their caregivers stay for six nights or less, tourism agencies can work with the medical providers to develop specifically designed services for the caregivers and the patients. This may include extra nursing support from the medical facility housing the patient, thus freeing up time and mental capacity for the caregivers of the patients to engage in more traditional tourism activities. For example, Children’s Hospital in Birmingham, AL., provides caregivers with a “parents night out,” along with coupons and a map to local attractions. Similarly, programs designed to bring local food and beverage to both the patient and the caregivers inside the medical facility could also be developed and packaged as an “add-on” when booking the medical tourism trip. Programs that offer assisted tourism activities to accommodate recovering patients and their caregivers may also be a worthwhile investment. Specialized transportation to accommodate wheelchairs and other mobility issues, the ability to skip long lines at popular tourism destinations, private tours that would allow for a nurse to join in on the excursion, and even bringing cultural or history-based lessons and programs to the medical facility are all practical ways to help ensure that both the patient and the caregivers themselves can fully engage with their destination from a tourism perspective. Furthermore, providing in-house travel planners at medical facilities which serve a large number of medical tourists may also be useful in this regard.

As noted earlier, a decreased length of stay may contribute to a less fulfilling and trustful relationship between patients and the doctors providing medical treatments, which may help explain why patients with the lowest length of stay did not see a supported path between the quality of medical care and the value for money. While the mean score of this sub-group was the second lowest out of the four (4.49), it was still relatively high considering that a five-point scale was used. Medical providers in destination countries may want to extend the contact time with the doctors and nurses providing the care, primarily via telehealth both before and after the physical manifestation of travel. This may also allow for the medical personal to interact with and respond to questions from a higher number of the patient’s caregivers (family and friends). Thus, simulating the experience of traveling with a larger group that consequently had a lower mean score for perceived medical care (4.36) but still supported the path between the quality of medical care and value for money. This mode of interaction may have also become more accepted by patients and medical providers due to the current Covid-19 pandemic.

### Theoretical implications

From a theoretical perspective, the current study has provided a substantive argument for using more nuanced measures when it comes to DI and medical tourists. The application of halo effect theory and the importance of post-visit evaluations of outbound medical tourists from a single country to different destinations has also added to the current body of knowledge. The uniqueness of the findings, especially based on LOS and TGS, may need further investigation, not only regarding why LOS and TGS seem to be such a differentiator for Bangladeshi medical tourists, but if the same finding would also be found in other groups that also participate in medical tourism.

### Limitations

This study utilized a non-probability convenience sampling technique to collect the necessary data via a paid consumer panel. As such, the results of this study cannot be generalized to the population of Bangladeshi outbound medical tourists. In addition, there was a lack of female respondents (only 9%) in the completed sample, which certainly brings into question the representativeness of the sample. A request was made to check for non-response bias. Several follow-up phone calls were made to women who did not respond to the survey. It was revealed that many of them did not feel comfortable answering the survey as they were not the primary decision-makers in choosing the medical tourism destination. In Bangladesh, men are overpoweringly the primary decision makers for large health-related purchases [78].

## Conclusion

This study has identified that subgroups within the Bangladeshi medical tourism customer base do exist and that these groups are examining the medical tourism product differently. These differences are essential because these groups are also flowing through the four components of DI to their re-visit intentions differently. The two subgroups, as identified by either their length of stay or the number of travelers accompanying the patient, represent opportunities for specialized marketing and services development, with the intent of maximizing their re-visit intentions.

## Supporting information

S1 File (SAV)
